# Green Processing of Neat Poly(lactic acid) Using Carbon Dioxide under Elevated Pressure for Preparation of Advanced Materials: A Review (2012–2022)

**DOI:** 10.3390/polym15040860

**Published:** 2023-02-09

**Authors:** Stoja Milovanovic, Ivana Lukic, Gabrijela Horvat, Zoran Novak, Sulamith Frerich, Marcus Petermann, Carlos A. García-González

**Affiliations:** 1 Faculty of Technology and Metallurgy, University of Belgrade, Karnegijeva 4, 11000 Belgrade, Serbia; 2 Łukasiewicz Research Network—New Chemical Syntheses Institute, Al. Tysiąclecia Państwa Polskiego 13a, 24-110 Puławy, Poland; 3 Faculty of Chemistry and Chemical Engineering, University of Maribor, Smetanova 17, SI-2000 Maribor, Slovenia; 4 Faculty of Mechanical Engineering, Institute of Thermo and Fluid Dynamics, Ruhr-University Bochum, Universitätsstraße 150, 44801 Bochum, Germany; 5 I+D Farma Group (GI-1645), Department of Pharmacology, Pharmacy and Pharmaceutical Technology, Faculty of Pharmacy, iMATUS and Health Research Institute of Santiago de Compostela (IDIS), Universidade de Santiago de Compostela, E-15782 Santiago de Compostela, Spain

**Keywords:** aerogels, drying, foaming, impregnation, particle generation, PLA

## Abstract

This review provides a concise overview of up-to-date developments in the processing of neat poly(lactic acid) (PLA), improvement in its properties, and preparation of advanced materials using a green medium (CO_2_ under elevated pressure). Pressurized CO_2_ in the dense and supercritical state is a superior alternative medium to organic solvents, as it is easily available, fully recyclable, has easily tunable properties, and can be completely removed from the final material without post-processing steps. This review summarizes the state of the art on PLA drying, impregnation, foaming, and particle generation by the employment of dense and supercritical CO_2_ for the development of new materials. An analysis of the effect of processing methods on the final material properties was focused on neat PLA and PLA with an addition of natural bioactive components. It was demonstrated that CO_2_-assisted processes enable the control of PLA properties, reduce operating times, and require less energy compared to conventional ones. The described environmentally friendly processing techniques and the versatility of PLA were employed for the preparation of foams, aerogels, scaffolds, microparticles, and nanoparticles, as well as bioactive materials. These PLA-based materials can find application in tissue engineering, drug delivery, active food packaging, compostable packaging, wastewater treatment, or thermal insulation, among others.

## 1. Introduction

Poly(lactic acid) (PLA) is a synthetic aliphatic polyester produced by the polymerization of lactic acid derived from renewable resources (plants as feedstock), such as agricultural waste and by-products, as a cheap and abundant source of lignocellulosic biomass [1]. It is extensively tested both at academic and industrial levels due to non-toxic components for its manufacturing as well as its biocompatibility, biodegradability, and/or compostability [2,3,4,5,6]. The emerging bio-based applications of materials coupled with the need of society to replace conventional plastics, as well as regulations that restrict plastic usage, encourage growth in the number of PLA-based products available to consumers. Several companies are working intensively towards the development of new and/or improved PLA types (Table 1).

NatureWorks is one of the world’s leading manufacturers of different types of PLA-based resins, with a capacity of 150,000 t/y [7]. TotalEnergies Corbion has a global marketing and sales network. Its facility in Rayong (Thailand) operates a capacity of 75,000 t/y [8]. This company has successfully produced PLA resin from alternative, second-generation feedstock, obtaining the same properties as current commercially available PLA resins. Evonik is a global leader company in PLA biomaterials for medical implants [9], while ThyssenKrupp helps the bioplastics’ PLA achieve its breakthrough in the food industry as beverage bottles [10]. With the rising demand, the costs of bioplastics have been continuously decreasing over the last decade [11]. Novel biorefinery concepts and policies concerning lactic acid production from agricultural waste and by-products, as well as newly developed processing methods that increased production and broader PLA commercial applications, should further decrease the price of PLA [7]. The current market price of PLA is 1.4–3.16 USD/kg [12,13].

Although PLA has good properties, including processability, its mass production and utilization are currently restricted due to low melt strength, slow crystallization, poor thermal resistance, brittleness, and/or sensitivity to moisture [2,5,14,15]. PLA treatment using CO_2_ under elevated pressure was suggested to improve material properties, endow material with new functionalities, or prepare material for a specific application [16,17]. Both dense (d-CO_2_) and supercritical CO_2_ (sc-CO_2_) were successfully used for PLA processing. Sc-CO_2_ is at conditions above its critical pressure (*P*_c_) of 7.38 MPa and critical temperature (*T*_c_) of 31.1 °C [18,19,20,21], whereas d-CO_2_ is at conditions close to but below its *P*_c_ and/or *T*_c_. The main advantage of d-CO_2_ and sc-CO_2_ utilization in PLA processing is that they are an alternative green media to environmentally harmful solvents currently used in the industry. D-CO_2_ and sc-CO_2_ are inexpensive, non-flammable, chemically inert with zero ozone-depletion potential, and generally regarded as having a safe status (GRAS) [4,5,18,20]. The versatility of CO_2_ under an elevated pressure for PLA processing is due to its special properties such as tunable solubility and diffusivity, which significantly increase with the change of CO_2_ from gas, liquid, and dense to a supercritical state [5]. Sc-CO_2_ can be dissolved in amorphous and semicrystalline polymers resulting in polymer plasticization, i.e., the increased segmental and chain mobility lowering their resistance to rotation, decrease in crystallization temperature (*T*_cr_), and decrease in glass transition temperature (*T*_g_) [2,5,15,18,19,22]. The Lewis acid–base interaction between CO_2_ and carbonyl groups as well as the C–H∙∙∙O hydrogen bonding facilitate the interaction of PLA with CO_2_ under elevated pressure [11,23,24]. When compressed, small changes in the process pressure and temperature can significantly influence the CO_2_ interaction with PLA. Moreover, due to the fact that CO_2_ is a gas under ambient conditions, its removal from the final product is easy, complete, and performed by venting, which avoids the post-processing for solvent removal [18].

**Figure 1:**
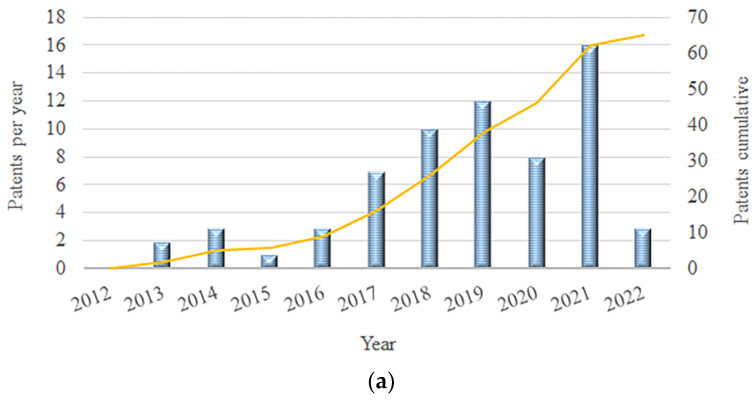

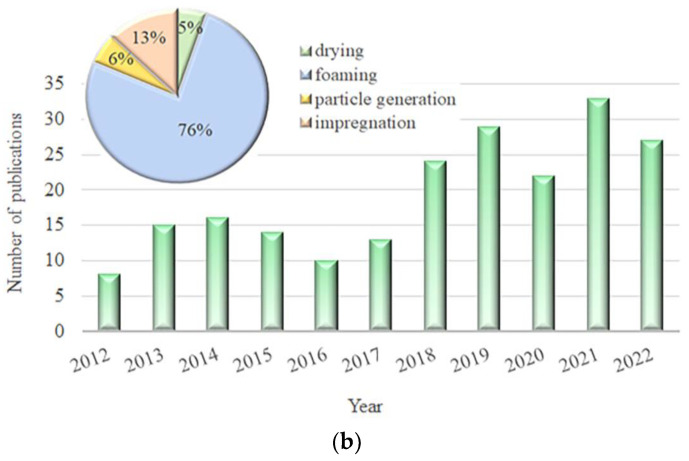
State-of-the-art and state-of-technique data on PLA processing for the 2012–2022 period: (**a**) patent survey and (**b**) survey on published research reports based on the Web of Science database with methods of PLA processing (Keywords: (“PLA” or “poly(lactic acid)”) and (“supercritical CO_2_” or “high-pressure CO_2_” or “dense CO_2_”) and (“drying” or “foaming” or “impregnation” or “particle generation” on 15 November 2022).

The growing interest in d-CO_2_ and sc-CO_2_-assisted methods as environmentally friendly technologies for PLA processing and the preparation of advanced materials is evident from the available patents and scientific literature published in the last decade. The patent survey by Espacenet for PLA processing using CO_2_ under elevated pressure, for the period 2012–2022, revealed 65 patents (Figure 1a). To include all processing techniques of interest for the present manuscript as accurately as possible, the keywords used were “PLA” or “poly(lactic acid)” and “supercritical CO_2_” or “high-pressure CO_2_” or “dense CO_2_” and “drying” or “foaming” or “impregnation” or “particle generation”. The highest number of patents (over 70%) as well as the fastest-growing trend was observed in the domain of PLA foaming. The graph presented in Figure 1b was based on a scientific literature survey of the Web of Science database for the same period and the same keywords. It showed a similar trend with a gradual increase in the number of publications by the year 2017, after which the number of publications rapidly increased. Among 211 reports, 76% are in the domain of foaming, confirming the high scientific interest in PLA processing into foams, which appears to be both increasingly attractive and the most promising new material option.

Several review articles from the available literature have suggested PLA processing using CO_2_ under elevated pressure [2,5,16,17,19,25,26] and, as mentioned, almost all of them are focused on foaming. However, the present article is the first systematic review of the process mechanisms, fundamental properties, and up-to-date technologies of drying, foaming, impregnation, and particle generation applied for the processing of PLA using d-CO_2_ and sc-CO_2_. This article also discusses the effect of process parameters on the application of the obtained PLA materials. A short overview for processes of interest and properties of the obtained advanced PLA materials is given in Table 2. Generally, this review is intended to stimulate further research on the application of green processing media (d-CO_2_ and sc-CO_2_) for polymer science and the engineering of advanced materials.

## 2. Drying of PLA Solutions, Gels, and Emulsions

Solvent-free porous PLA material can be obtained by the drying of solutions, gels, or emulsions by the removal of a liquid solvent. This can be performed either by evaporative drying, freeze-drying, or supercritical drying. Evaporative (air) drying at an ambient pressure results in xerogels with collapsed pores as a consequence of the vapor–liquid surface tension forming inside the wet matrix [27,28], while freeze-drying is time-consuming and may lead to a solvent crystal growth during freezing and, consequently, pore growth that develops stress inside pores and leads to fractures [29,30]. Supercritical drying techniques overcome the problems encountered with traditional drying methods and preserve the high and open porosity of wet materials in a dry form [31]. To decrease the vapor–liquid surface tension, increase the rate of drying, or remove potentially toxic solvents, the replacement of a solvent contained in a polymer matrix with a non-solvent (such as ethanol or acetone) prior to the supercritical drying is suggested [32]. Supercritical drying allows the solvent or non-solvent extraction from a wet material through the formation of a supercritical mixture between CO_2_ and liquid present in the polymer matrix. The supercritical mixture shows no surface tension and can be easily eliminated by system venting [33]. The rate of solvent/non-solvent removal from a polymer matrix depends on numerous parameters [34]. Two of the most important are solvent/non-solvent solubility in sc-CO_2_ and diffusivity in a polymer matrix. For instance, dioxane has high solubility, and it was reported that the amount of remaining dioxane in the PLA scaffold was 263 ppm after 4 h and only 5 ppm after 6 h of sc-CO_2_ drying [35].

### 2.1. Methods for Sc-CO_2_-Assisted Drying of Wet PLA

**Figure 2:**
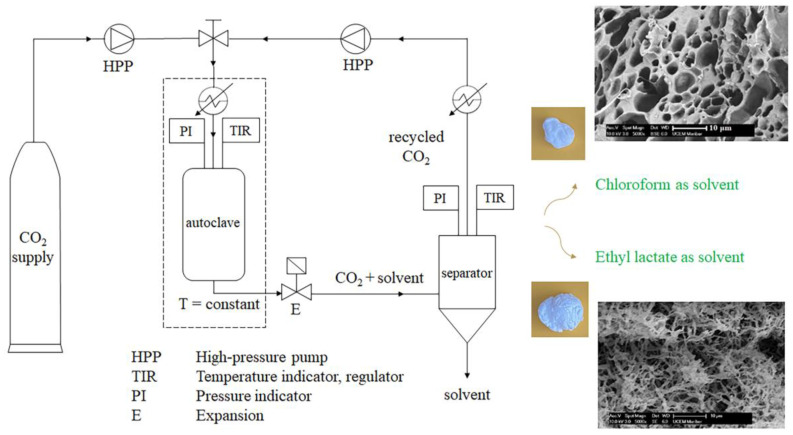
Schematic presentation of high-pressure unit used for dynamic supercritical drying with CO_2_ recycling with images of obtained PLA aerogels.

The drying of wet PLA materials using sc-CO_2_ can be roughly divided into static and dynamic methods. The static method implies the contact of wet material and sc-CO_2_ at constant pressure and temperature without the introduction of “fresh” CO_2_ into the vessel. Reverchon et al. [35] applied static sc-CO_2_-drying for the preparation of PLLA scaffolds and aerogels with a porosity of 95% at 20 MPa and 33 °C. They reported that an increase in pressure increases the sc-CO_2_ solvation power, enabling a faster solvent elimination, and that an increase in operating time from 4 to 8 h significantly affects the solvent (dioxane) elimination, pinpointing 6 h as the optimal time [35]. Dynamic drying implies applying a constant flow of “fresh” sc-CO_2_ through the vessel (Figure 2). It was reported that dynamic supercritical drying is beneficial to the environment and is thriftier in energy consumption compared with the classical freeze-drying process. Various life cycle assessment methodologies indicated the possible reduction in the environmental impact, as between 50 and 90% were attributed to the supercritical process [36]. A combination of a static and dynamic supercritical drying (SCD) of wet PLA was also reported, where after a certain time in the discontinuous mode, sc-CO_2_ enriched with the solvent/non-solvent of a gel is replaced with fresh sc-CO_2_ by applying its continuous flow through the vessel for a certain time [37,38,39]. This combined process can be run for several cycles, while at the same time maintaining a constant process pressure and temperature.

There are several variations on supercritical techniques for drying wet PLA reported in the literature such as supercritical drying combined with thermally induced phase separation (STIPS) [36], supercritical freeze extraction process (SFEP) [40,41], supercritical drying combined with particle leaching (SPL) [35,39], and supercritical emulsion extraction (SEE) [42,43,44] (Table 3).

Thermally induced phase separation (TIPS) implies cooling the PLA solution to a temperature, where the solvent and PLA are not miscible to trigger the phase separation. It is one of the most effective processes for the production of a material with tunable biodegradability, mechanical properties, porosity, pore diameters, and pores’ interconnectivity [36]. The morphology of PLA material can be controlled by adjusting the TIPS process conditions such as the polymer concentration and molecular weight, the selection of solvent, the cooling rate, and temperature during solvent replacement [36,37]. The STIPS process refers to TIPS, after which the supercritical drying is applied. Salerno and Domingo [33] modulated the material porosity from 95 to 90% and the specific surface area from 69 to 95 m^2^/g by increasing the PLA concentration in a solution with ethyl lactate from 3 to 5.5 wt%, which was followed by solvent replacement (first with water and afterward with ethanol) prior to STIPS. This increase in concentration also increased the mean fiber diameter of aerogels from 100 to 200 nm and a pore volume fraction from 0.044 to 0.055 cm^3^/g. Solvent selection is an additional parameter that significantly affects the morphology of the final PLA material [17], as can be observed in Figure 2. The choice of a solvent is primarily important for the complete dissolution of a polymer. Selection of an appropriate solvent for PLA is relatively limited and eliminates most of the more environmentally friendly solvents that have good solubility in sc-CO_2_ used in the drying process (such as ethanol). It was previously reported that among the tested solvents (ethyl lactate, tetrahydrofuran, chloroform, dioxane, and dichloromethane), the highest PLA material porosity (73%) was obtained using chloroform [37]. Differences between obtained materials can be explained by the differences in the individual Hansen solubility parameters, the viscosity of the obtained solutions, and the rate of solvent evaporation [45]. Although miscibility between PLA and chloroform or dichloromethane is high, dichloromethane has Hansen parameters most similar to PLA. Moreover, the PLA solution with chloroform had a higher viscosity compared with the solution with dichloromethane [45]. Finally, dichloromethane evaporates faster compared with chloroform. In addition, it was previously reported that PLA-dichloromethane solutions can lead to the polymer chain entanglement and the physicochemical interaction between the solvent and PLA. An additional parameter to control the sc-CO_2_ drying of polymers with low *T*_g_ is the polymer crystallinity degree [46]. Namely, PLA properties vary significantly, depending on its isomers’ (poly(L-lactic acid) (PLLA), poly(D-lactic acid) (PDLA), and poly(D,L-lactic acid) (PDLLA)) content [47,48,49,50]. PLA with L-content higher than 93% tends to crystallize, while PLA with L-content in the range from 50 to 93% is amorphous [2,51,52]. Bueno et al. [46] tested the gelation of semicrystalline PLLA and amorphous PDLLA using the solvents chloroform + methanol or DMSO + ethanol, TIPS, ethanol as a replacement solvent, and sc-CO_2_ drying at 40 °C and 10 MPa for 1 h [46]. They concluded that the STIPS resulted in the structural collapse of amorphous polymers in all cases, while a minimum crystallinity degree of 25% combined with a second non-solvent exchange with liquid CO_2_ before the drying process allowed the preparation of aerogels with a surface area up to 85 m^2^/g. It was also reported that the PLLA concentration of about 5 wt% and cooling temperature below −80 °C favored the formation of open pore structures with the scaffold’s surface connectivity, while an increase in the PLLA molecular weight induced a significant decrease in the pores’ diameter, from 10 to 1 μm [36].

The SFEP process combines the advantages of TIPS [39,53,54] and immediate supercritical drying. The process was successfully used to generate 3D PLA scaffolds characterized by a micrometric cellular structure and wrinkled pores’ walls, with porosity ranging between 93% (for 5% *w*/*w* PLLA scaffold) and 84% (for 20% *w*/*w* PLLA scaffold) [40]. It was reported that an increase in PLA concentration from 5 to 20% *w*/*w* led to a decrease in the mean pore size (from about 15 to 3 μm) and pore size distribution.

The SPL process implies particle leaching from a material that was initially dried using the supercritical fluid. The SPL technique helps in achieving a fine control over the porous scaffold architecture network by the selection of the appropriate concentration and the size of the particulate porogen [39]. Reverchon et al. [35] prepared PLA scaffolds with elevated porosities to 97.2%, surface areas of 45 m^2^/g, and good mechanical properties (compressive modulus up to 81 kPa) in the formation of a PLA gel loaded with fructose, sc-CO_2_ drying, and washing with water to eliminate the porogen. It was also reported that the combination of TIPS with SPL techniques can improve the preparation of PLA scaffolds [39]. This three-step process aimed to induce an additional interconnected network and create the nanometer-scale fibrous architecture combined with large pores of the PLA matrix.

**Table 3:** Literature reports on the drying of PLA solutions, gels, and emulsions using sc-CO_2_ (2012–2022).

Material	Producer	Method	Regime	*P* (MPa)	*T* (°C)	*t* (h)	Material Properties	Material Application	Lit
PLLA	Purac Biochem	STIPS	Static	19	39	1.5	*ε* = 90–95% *S* = 70–95 m^2^/g	Tissue engineering scaffolds	[33]
2500HP 4060D	Natureworks	STIPS	Dynamic	10	40	1	*S* = 29–85 m^2^/g	Pharmaceutical	[46]
PLLA	Purac Biochem	STIPS SPL	Static	19	39	1.5	*ε* = 79–98% *D*_ave_ = 171–440 μm *S* = 29–39 m^2^/g	Tissue engineering scaffolds	[39]
PLLA with ibuprofen	Boehringer Ingelheim	SFEP	Static	10–20	35–55	4	*ε* = 78–89% *R*_aver_ = 8–30 μm *D*_aver_ = 8–30 μm	Tissue engineering scaffolds	[40]
PLLA	Boehringer Ingelheim	SFEP	Static	10–25	35	4	*ε* = 84–93% *D*_aver_ = 3–15 μm	Tissue engineering scaffolds	[41]
3052D	NatureWorks	SCD STIPS	Static and dynamic	15, 19	35, 39	2.7	*ρ* = 337–468 kg/m^3^ *ε* = 63–73% *D*_aver_ = 0.08–5.1 μm	Wastewater treatment	[37,38]
PLLA	synthetized	STIPS	Dynamic	15	35	4	*ρ* = 75–310 kg/m^3^ *ε* = 75–94% *D*_aver_ = 3–138 μm	Tissue engineering scaffolds	[36]
203H	Evonik Industries	SEE	Dynamic	8	38	n.i.a.	*R*_aver_ = 0.2–0.9 μm	Bactericidal nanocomposites	[42]
708H	Evonik Industries	SEE	Dynamic	8	38	n.i.a.	*R*_aver_ = 1.5 μm	Nutraceutical formulation	[43]
203H	Evonik Industries	SEE	Dynamic	8	39	n.i.a.	*R*_aver_ = 0.4–3 μm	Growth factor delivery	[44]

*P*—pressure; *T*—temperature; *t*—time; *ε*—porosity; *S*—surface area; *D*_aver_—average pore diameter; *R*_aver_—particle mean size; *ρ*—bulk density; n.i.a.—no information available.

The SEE technique is a continuous process that involves the countercurrent extraction of a solvent in the oily phase of the emulsion (the emulsion is introduced at the top of the column while sc-CO_2_ is introduced from the bottom). A refrigerated separator is located downstream of the column for the collection of the oily phase. Another separator is located at the bottom of the column to collect the particles suspended in water [43,44]. During the evaporation or extraction of the emulsion, the oily droplets dispersed in the water external phase are hardened, leading to the formation of micro/nanoparticles [44]. This method, reported for the preparation of micro and nano-carriers, has been described as excellent given its mild processing conditions and easier solvent removal. SEE assured an improved batch-to-batch reproducibility and accurate carrier size control thanks to a fixed droplet shrinkage without aggregation phenomena, lower solvent residue, and controlled drug encapsulation efficiency [44]. SEE at 8 MPa and 45 °C in a packed column was used for the efficient elimination of the benzyl alcohol residue from lipid nanoparticles [42]. When the nanosuspension was treated with sc-CO_2_, fast and complete elimination of the residual benzyl alcohol led to particle disaggregation and the formation of nanoparticles with a strongly reduced mean diameter. The SEE technology enabled the preparation of PLA carriers with the advanced control of their size and morphology. It was also reported that carriers with different mean sizes from 0.4 to 3 μm can be obtained by the removal of ethyl acetate from emulsions at 8 MPa and 39 °C [44].

Based on the results presented in the available literature on the drying of wet PLA, it can be concluded that the selection of an sc-CO_2_ drying regime, operating pressure, time, selection of PLA type, solvent for PLA, PLA/solvent ratio, addition of porogen, and addition of antisolvent significantly affect the final material properties and its application. The influence of temperature was only rarely studied, since it had to be as low as possible while maintaining supercritical conditions. However, this technique is challenging to apply for the drying of PLA gels if the polymer has low *T*_g_, which further decreases in the presence of sc-CO_2_ [46]. Namely, if the processing temperature during sc-CO_2_-assisted drying is above the polymers’ *T*_g_, it may result in the gel collapse producing the final material with a low surface area [39,46]. Therefore, the operating temperature for sc-CO_2_ drying in all reported studies (Table 3) was the lowest possible to maintain CO_2_ at supercritical conditions and at the same time to avoid crossing the *T*_g_ of PLA.

### 2.2. Application of Dried PLA Materials

Highly porous PLA could be used in several applications, from wastewater treatment to tissue engineering or drug delivery. PLA aerogels can be designed with various morphologies (monoliths, beads, particles, etc.), mesoporous and/or microporous structures, large surface areas, and large porosity. It was reported that aerogels possess unique transport, adsorption, structural, and biophysical properties suitable for technological and biomedical applications [33,55]. The high and open porosity of aerogels is of significant relevance for wound healing, as it allows a gas permeability, avoiding hypoxia episodes and allowing transpiration and/or evaporation equilibrium at the wound site as well as a high liquid absorption capacity [55]. It is interesting to notice that PLA aerogels usually have lower surface areas (below 100 m^2^g^−1^) compared with other organic aerogels (200–300 m^2^g^−1^), possibly due to the low mesoporous volume that is either lost during the gelation or during the supercritical drying [46]. Generally, aerogel scaffolds have a 3D structure that can provide mechanical integrity and an appropriate surface to support regenerating tissues [19,55]. The key to tissue engineering is to temporarily surrogate the biological tissue as a replacement for diseased or damaged tissues with immunologically tolerant living/viable tissue that can grow with the patient [19]. Being able to control the pore size and its distribution by variation in parameters of CO_2_-assisted processes is essential in creating scaffolds and aerogels suitable for tissue engineering [56]. An important parameter of PLA aerogels is that they are biocompatible and have mechanical properties similar to cancellous bone, making them suitable for tissue engineering applications [57]. It was reported that biomimetic PLA scaffolds, consisting of interconnected networks with nanometer-scale fibrous architecture and pores in the range of 100–200 μm and 400–600 μm, are highly suitable for cell colonization and migration [39]. PLA aerogels could also be prepared in the form of microparticles as nutraceutical formulations (i.e., carriers of bioactive components such as *β*-carotene, *α*-tocopherol, and rosmarinic acid that exhibit high antioxidant activity) [43]. In addition, porous PLA (pores’ mean size from 0.4 to 3 μm) provided the sustained release of a growth factor over 25 days without intrinsic toxicity, revealing that they can be safely introduced within biomedical structures [44].

## 3. PLA Foaming Using CO_2_ under Elevated Pressure

Polymer foam is defined as a gas–polymer two-phase system, a material whose matrix contains a large number of macro, micro, and/or nanosized pores [58]. Although PLA foaming can be performed using organic blowing agents, their traces in foams could be harmful to material end-users [5]. Therefore, the replacement of organic with physical blowing agents such as CO_2_ under high pressure gained significant scientific attention. PLA foaming using d-CO_2_ or sc-CO_2_ can be performed in several steps: (1) exposure of PLA to CO_2_ at a certain pressure and temperature that allows the diffusion and/or dissolution of CO_2_ into the polymer matrix (diffused and/or dissolved CO_2_ increases the intermolecular distance causing polymer swelling, despite the hydrostatic pressure effect), (2) induction of a pressure drop or a temperature increase that cause cell nucleation and growth due to the thermo-dynamic instability from the supersaturation of CO_2_, and (3) separation of CO_2_ from the PLA-CO_2_ mixture and stabilization of the foam structure by cooling [2,5,18]. Setting the pressure and temperature conditions for the treatment of the polymer–CO_2_ mixture, to lock in the desired properties as cells develop during the phase separation, is essential for the generation, growth, and connectivity of pores [15,22]. As the pressure increases, causing a change in the CO_2_ state from dense to supercritical, more CO_2_ molecules dissolve in the PLA matrix, increasing the volume of the PLA/CO_2_ mixtures. In other words, the dissolved fluid increases the intermolecular distance, causes swelling, and consequently, both the free volume and the volume of the polymer/fluid mixture increase. In addition, when the temperature rises, the PLA’s molecular movement further increases. Thus, the free volume and the specific volume increase. In addition, if the decompression rate is high, nucleation is rapidly leading to a large number of nucleation sites. Each pore will develop so fast that the CO_2_ diffusion will be negligible and the resulting material will have a uniform distribution of small pores [19]. On the other hand, if the nucleation is very slow, the pores that nucleated first will be significantly larger compared with later-formed others, due to the diffusion of CO_2_ from the surrounding polymer matrix. The resulting material will have a wide distribution of pore size [19].

Foaming is highly influenced by the solubility of CO_2_ in PLA, which depends on employed process conditions (pressure and temperature), the affinity of CO_2_ towards polymer, and polymer chemistry [59,60,61,62,63,64]. Mahmood et al. [60] tested the solubility at the pressure range of 6.9–21 MPa and temperature range of 80–100 °C, concluding that the CO_2_ solubility in PLA was 0.096 g/g achieved at 20.7 MPa and 80 °C. They also demonstrated that an increase in temperature increased PLA swelling and decreased CO_2_ solubility, whereas an increase in pressure increased both PLA swelling and CO_2_ solubility. Li et al. [61] tested the solubility at a wider pressure and temperature range (3.5–28 MPa and 180–200 °C, respectively), concluding that the CO_2_ solubility in PLA was 0.104 g/g achieved at 28 MPa and 180 °C. The CO_2_ solubility in semi-crystalline PLA is more complex compared with amorphous PLA because compressed CO_2_ can accelerate the crystallization process [5,65,66,67]. Moreover, the presence of crystals decreases the CO_2_ solubility in the PLA matrix and dramatically affects cell nucleation and cell growth [59,65,68].

### 3.1. Methods and Parameters for PLA Foaming under High-Pressure CO_2_

**Figure 3:**
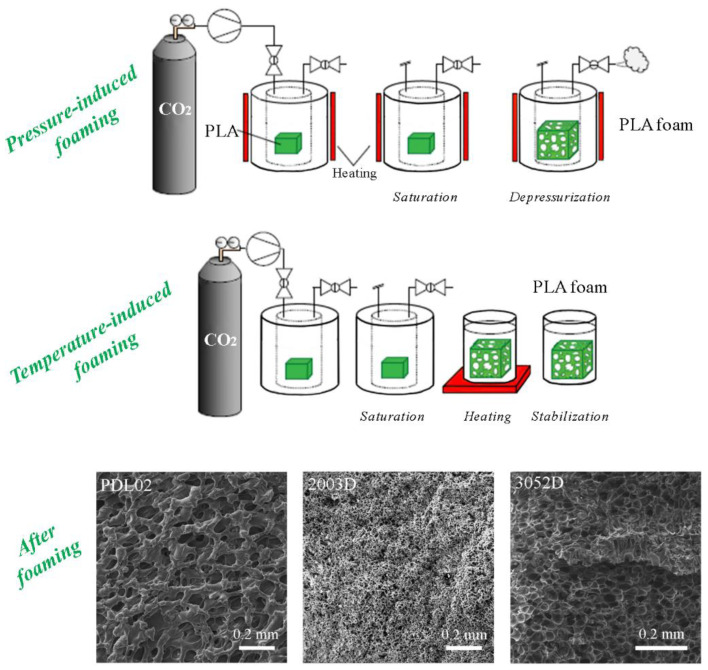
Simplified presentation of the pressure-induced and temperature-induced batch foaming (adapted from [16]) with SEM images of obtained foams.

PLA foaming using CO_2_ under an elevated pressure can be performed as pressure-induced or temperature-induced foaming in a static (batch) or continuous regime (Figure 3) [2]. When a thermodynamic instability is caused by the opening of an outlet valve, the pressure drops, the heated polymer becomes over-saturated and it cannot retain the previously dissolved CO_2_ [2]. Then, phase separation occurs and cell nucleation and growth take place, leading to the expansion of PLA and the formation of a porous structure. In the case of temperature-induced foaming, thermodynamic instability is caused by the immersion of a polymer with CO_2_ under elevated pressure in hot water, glycerin, or oil. Foaming is initiated when the applied temperature increase leads to increased chain mobility as the polymer is softened and CO_2_ solubility decreases, which results in cell nucleation and growth. The final cooling step ensures the stabilization of the foam [16,51].

A literature survey on the PLA foaming process during the last decade is shown in Table 4. Generally, it was reported that PLA foaming is significantly affected by the process parameters, i.e., pressure, temperature, soaking time, and depressurization rate [65,68,69,70,71,72]. It was reported that an increase in pressure during sc-CO_2_ foaming increases PLA expansion ratios [71]. At higher pressures, the amount of CO_2_ incorporated into the polymers is greater, and hence the polymer is highly supersaturated upon decompression. These greater supersaturation pressures lead to higher nucleation densities and hence smaller pores [73]. Kiran [69] reported that smaller pores were promoted when PLA foaming was carried out by depressurization from higher pressures. Several reports from available literature state that lower temperatures lead to the generation of smaller pores during the sc-CO_2_ foaming process [69,73]. This observation is consistent with the notion of a greater level of CO_2_ incorporation within PLA due to the increased density and solubility with a decreasing temperature at constant pressure, which is followed by the formation of a greater number of nucleation sites during decompression. For instance, lowering the process temperature from 55 to 35 °C at 23 MPa decreased the pore diameter from around 200 to 50 μm [73]. Yan et al. [72] reported that an increase in temperature from 130 to 160 °C at 20 MPa increases the PLA cell diameter from 0.2 to 110 μm and decreases the cell density from 10^13^ to 10^3^ cells/cm^3^. Yang et al. [65] reported that the crystallinity of PLA foams decreased with the increasing annealing temperature or pressure, while crystal morphologies were varied by controlling the temperature or pressure in the range of 70–120 °C and 8–24 MPa, respectively. Haham et al. [71] reported that an increase in soaking time from 5 to 20 min at 33 MPa resulted in a decrease in the mean pore size from 117 to 11 μm and an increased cell density up to 4 orders of magnitude. The soaking time mainly affects the distribution of d-CO_2_ or sc-CO_2_ through a polymer matrix. When a soaking time is insufficient, it will result in heterogeneous structures. On the other hand, the prolongation of soaking allows the dissolution of a higher amount of d-CO_2_ or sc-CO_2_, which leads to the formation of foams with higher cell densities and reduced pore diameters [74].

Besides process conditions, PLA foaming is affected by the selection of PLA type. It was reported that PLA has a molecular weight (*M*_w_) in the range of 10–258 kg/mol, *T*_g_ in the range of 33–65 °C, and *T*_m_ in the range of 100–200 °C [17], which can determine the selection of process parameters and, consequently, the foam morphology (Figure 3). It was reported that foams of PLA (PDL02, *M*_w_ = 17 kg/mol) can be obtained at a lower pressure and temperature condition (10–15 MPa and 35–50 °C after a soaking time of 2–6 h) [75]. Kuska et al. [15] reported that already at pressures below 5 MPa, d-CO_2_ present in the polymer matrix acted like a molecular lubricant. It enhanced the chain mobility by weakening intermolecular and intramolecular interactions and reduced *T*_m_ and *T*_sr_ as well as the melt viscosity of PLA. However, only at temperatures equal to or higher than 100 °C, foams of PLA with higher *M*_w_ (3052D of 160 kg/mol and 2003D of 210 kg/mol) could be obtained. For instance, PLA (2003D) foams with a porosity of 55–66%, density of 426–685 kg/m^3^, and average pore size of 15–200 μm could be obtained at pressures of 20–30 MPa and temperatures of 100–120 °C after 2 or 24 h of soaking [15].

PLA foaming with d-CO_2_ or sc-CO_2_ can be aided with an organic solvent that affects the CO_2_ solubility in PLA [69,76]. The process can be performed by the (a) CO_2_ introduction into a vessel containing a PLA solution in an organic solvent, (b) PLA solution spraying into a vessel containing CO_2_, or (c) simultaneous spraying of a PLA solution and CO_2_ into a vessel [69]. Kiran [69] reported PLA foaming using sc-CO_2_ and acetone binary mixture at pressures in the range of 14–61 MPa. Even though PLA was not soluble in sc-CO_2_ at the tested conditions, PLA was completely solubilized in mixtures of CO_2_ and acetone, and foams with diameters of 10–20 μm were obtained [69].

**Figure 4:**
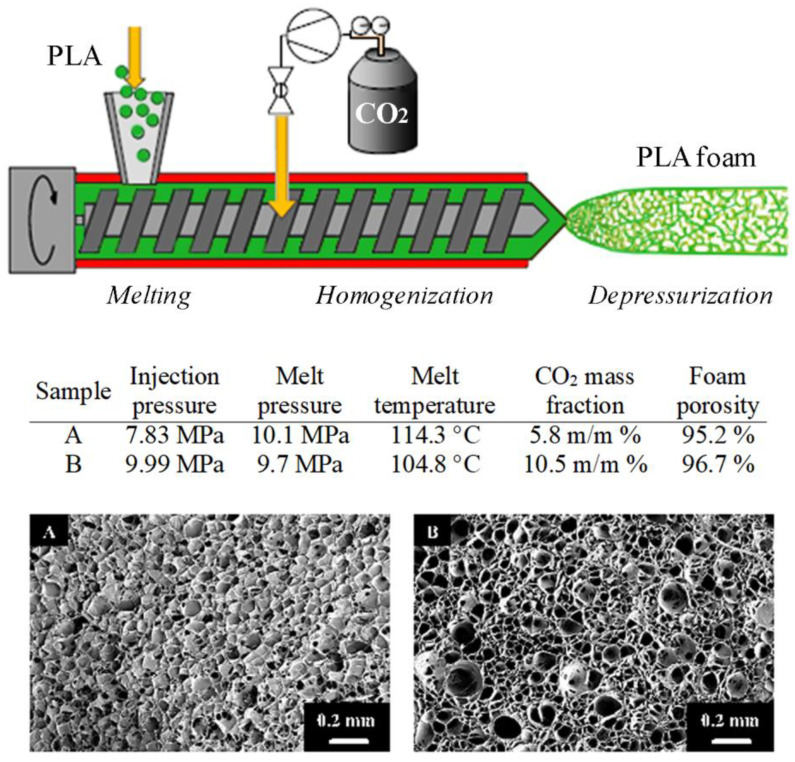
Simplified presentation of the sc-CO_2_-assisted extrusion foaming process (adapted from [16]), foaming conditions for PLA 2003D in the planetary roller extruder, and SEM images of corresponding foams.

One of the reported techniques for PLA foaming is CO_2_-assisted extrusion. The extrusion foaming process implies the injection of CO_2_ into the extruder barrel under the high pressure and continuous mixing of a PLA melt and CO_2_ inside the barrel to dissolve or homogenize the CO_2_ within the polymer melt [5]. The PLA-CO_2_ mixture will flow along the extruder and emerge from the die, where the induced pressure drop will cause CO_2_ separation from the melt and PLA foaming (Figure 4). One of the biggest advantages of this technique is its continuous work regime, which enables a relatively easy transition from the laboratory to an industrial level. The process is usually performed using d-CO_2_, due to higher investment costs and process difficulties associated with the sc-CO_2_-assisted extrusion, such as the pressure fluctuation inside the barrel at the injection point of CO_2_, maintaining sufficient CO_2_ pressure downstream, and prevention of injected CO_2_ from leaking upstream, as well as ensuring good mixing [18]. PLA extrusion foaming using CO_2_ as the blowing agent is marked as a 100% “green” technology that avoids the use of any organic solvent [26]. In addition, sc-CO_2_ can enable the production of microcellular foams with an average cell density above 10^9^ cells/cm^3^, which demonstrated improved fatigue life, toughness, thermal stability, and insulating properties compared with conventional foams [26]. The introduction of d-CO_2_ or sc-CO_2_ in the barrel of an extruder lowers the polymer viscosity and allows lower operating temperatures compared with the standard extrusion processes, which consequently leads to less energy consumption [77].

Pore sizes of PLA foams and their distribution are significantly affected by extrusion conditions (i.e., barrel temperature, number of temperature zones, number of screws, design of screws, screw speed, torque, CO_2_ inlet, die temperature, and die design) and mixture composition (i.e., PLA properties, CO_2_/PLA ratio, eventual presence of an additive, PLA/additive ratio) [2,26,78]. Chauvet et al. [78] found that the temperature before and inside the die was the most prominent parameter to tune the PLA foam properties. They demonstrated that foam porosity as high as 96% could be obtained when the die temperature was between 109 and 112 °C, and the CO_2_ injection pressure was between 19.7 and 23.8 MPa. In PLA foam extrusion experiments using a tandem-extrusion line consisting of a planetary roller extruder as a mixing and cooling extruder [79], similar results were reported for melt temperatures in the die between 103 and 114 °C, with lower CO_2_ injection pressures between 7.9 and 11.2 MPa. Foaming conditions and SEM images of these samples with resulting foam morphologies perpendicular to the extrusion direction are shown in Figure 4.

Lee et al. [80] reported a decrease in the PLA foam density from around 300 to 200 kg/m^3^ with a decrease in the die temperature from 140 to 120 °C. It was also reported that the crystallinity of PLA helps foam properties but negatively affects its processing. A trade-off between these two parameters must be kept for satisfactory foam production [80]. Larsen and Neldin [52] reported that not only the variation in die temperature but also the pressure drop rate can affect PLA foam properties. They suggested that the pressure drop rate should be around 1 GPa/s or higher to achieve foams with densities of 24–29 kg/m^3^, pore diameters of 14–31 μm, and cell densities of 2.0–9.0·10^8^ cell/cm^3^. In addition, a relatively high CO_2_/PLA ratio is required to sustain the fine-celled microcellular foam, because the smaller the bubble, the higher the surface tension force from the surrounding melt phase [80]. Reignier et al. [6] increased the CO_2_ concentration from 1.8 to 9.4 wt% in a PLA melt, achieving pressures from 10.2 to 20.7 MPa, which resulted in more than 50% viscosity decrease, a decrease in foam density to less than 30 kg/m^3^, increase in cell density to 10^8^ cell/cm^3^, and decrease in pore size below 100 μm.

**Table 4:** Literature reports on PLA foaming using high-pressure CO_2_ (2012–2022).

PLA	Producer	CO_2_	Regime Method	*P* (MPa)	*T* (°C)	*t* (h)	Foam Properties	Foam Application	Lit
PDL02	Corbion Purac	sc-CO_2_	Static	10, 15	35, 40, 50	2–24	*T*_g_ = 31 °C *D*_aver_ = 67 μm	Drug delivery	[75]
2003D	NatureWorks	sc-CO_2_	Static	20–30	100–120	2, 24	*ρ* = 427–685 kg/m^3^ *ε* = 45–66%	Food packaging	[15]
2002D	NatureWorks	sc-CO_2_	Static	20–30	131,132	0.08–12	*E*_c_ = 61–82 MPa *σ*_60_ = 9–11 MPa	n.i.a.	[70]
2003D	NatureWorks	sc-CO_2_	Static	8–24	70–120	1	*D*_aver_ = 4–23 μm *ε* = 43–80%	n.i.a.	[65]
2003D	NatureWorks	sc-CO_2_	Static	10–20	130, 190	1	*D*_aver_ = 0–200 μm	n.i.a.	[81]
L175 DO70	Total Corbion	sc-CO_2_	Static	15	45	8	*D*_aver_ = 2–4 μm *N*_c_ ~2∙10^10^ cells/cm^3^	n.i.a.	[82]
4032D	NatureWorks	sc-CO_2_	Extrusion and static	10–31	190 115–155	0.25	*D*_aver_ = 0.7–22 μm *N*_c_ ~10^11^ cells/cm^3^	Thermal insulation	[63]
4032D	NatureWorks	sc-CO_2_	Extrusion and static	17–31	190 118–143	0.03–1	*D*_aver_ = 1–22 μm *N*_c_ = 10^8^–10^12^ cells/cm^3^	Oil sorption	[64]
4060D	NatureWorks	sc-CO_2_	Extrusion and static	10–33	170–180 40	0.08–0.5	*D*_aver_ = 11–117 μm *N*_c_ = 10^3^–10^9^ cells/cm^3^	n.i.a.	[71]
2003D	NatureWorks	sc-CO_2_	Heat pressing and static	15	190 (85–117)	2	*D*_aver_ = 31–1700 nm *N*_c_ = 10^13^–10^15^ cells/cm^3^	Thermal insulation	[83]
4032D	NatureWorks	d-CO_2_ sc-CO_2_	Two-step static	6.9–17.2	180 (85–125)	0.67	*D*_aver_ = 25–287 μm *N*_c_ ~10^4^–10^7^ cells/cm^3^	Oil adsorption; thermal insulation	[68]
2003D	NatureWorks	d-CO_2_ sc-CO_2_	Extrusion	6–8	140–190	n.i.a	*D*_aver_ = 0.1–1 mm *χ* = 1.5–15.5%	n.i.a.	[84]
PLE001	NaturePlast	sc-CO_2_	Extrusion	17.7–23.8	100–180	n.i.a	*ε* = 55–96% *T*_m_ = 139.5–142.9 °C	Food packaging	[78]

*P*—pressure; *T*—temperature; *t*—time; *T*_g_—glass transition temperature; *T*_m_—melting temperature; *D*_aver_—average pore diameter; *ρ*—foam density; *N*_c_—cell density; *ε*—porosity; *E*_c_—compressive module; *σ*_60_—compressive strength that was needed to compress the sample cores at about 60% of their original lengths; *χ*—crystallinity; n.i.a.—no information available.

### 3.2. Application of PLA Foams

PLA foams have been considered substitutes for some petroleum-based foamed products due to their competitive material and processing costs, comparable mechanical properties, and environmentally friendly properties. They have a wide array of applications such as packaging, cushioning, construction, thermal insulation, sound insulation, plastic utensils, automotive, etc. [16,51,58,68,85].

CO_2_-assisted processing techniques for the preparation of PLA microcellular structures enable the improvement of material properties, saving material and energy, leading to a significant lowering of the production cost. The reason for such a versatility of PLA materials lies in their chemistry. For instance, PLA with high *M*_w_, high crystallinity, and hardness is suitable for the construction of bearing materials such as screws, plates, or scaffolds for use as orthopedic implants. On the other hand, PLA with low *M*_w_ and low crystallinity is suitable for the preparation of controlled drug release systems that require proper biodegradation characteristics and the incorporation of drugs [66]. In addition, the optimization of the PLA foaming process may open a wide range of opportunities for designing novel multi-functional materials and products.

Biodegradable PLA foams show great potential for oil adsorption and thermal insulation, which is crucial for oil-spill cleanup and energy saving [68]. The closed-cell foam, with an expansion ratio of 60, showed a thermal conductivity as low as 31.7 mW/m·k. The open-cell foam, with an expansion ratio of 43, showed a water contact angle of up to 123° and exhibited an adsorption capacity in the range of 10.9–31.2 g/g for various oils [68].

PLA foams can have excellent properties such as a controlled morphology and lightweight, which enable the release of bioactive components and drugs in a controlled manner [75]. They can also be used in tissue engineering, promoting cell adhesion, proliferation, and growth [63].

## 4. PLA Particle Generation

The preparation of PLA particles using CO_2_ under elevated pressure avoids issues associated with conventional methods for particle generations such as long processing time, unwanted particle morphologies, particle agglomeration, undesired amorphous or crystalline forms, etc. [86,87]. Techniques for the generation of PLA particles use d-CO_2_ and sc-CO_2_, both as a solvent and anti-solvent. Besides the solubility of sc-CO_2_ in PLA, the solubility of PLA in sc-CO_2_ is an important parameter for the selection of appropriate particle generation techniques. The solubility of PLA in sc-CO_2_ depends on its chemical structure as well as on its physicochemical and mechanical properties, e.g., molar mass, end groups, the architecture (branched or linear), *T*_g_, and crystallinity. However, it also depends on the experimental conditions, such as pressure, temperature, and the presence or absence of a co-solvent in the system. The solubility of PLA in sc-CO_2_ is limited, which was explained, among other factors, by its lower chain flexibility [88]. Nevertheless, the solubility of polyesters can be positively influenced, e.g., by decreasing its *M*_w_. It was demonstrated that a PLA with a relatively high *M*_w_ of 128 kg/mol was not soluble in sc-CO_2_ even at high-pressure conditions above 139.8 MPa [89]. However, it was possible to process PLA with a low *M*_w_ of 5.5 kg/mol in sc-CO_2_ at the pressure of 25 MPa and temperature of 65 °C without the addition of any co-solvents for particle generation [90]. Still, if the polymer remains insoluble in sc-CO_2_ even under high-pressure conditions, polar co-solvents such as ethanol or acetone can be used.

### 4.1. Methods for PLA Particle Generation Using CO_2_ under Elevated Pressure

Several spraying or precipitation CO_2_-assisted techniques could be employed for the preparation of PLA particles, such as Particles from Gas Saturated Solutions (PGSS), Rapid Expansion of Supercritical Solutions (RESS), Supercritical Assisted Atomization (SAA), Supercritical Anti-Solvent (SAS), Solution Enhanced Dispersion by Supercritical fluids (SEDS), among others.

The PGSS process allows the formation of particles in several steps: (1) the polymer is melted or dissolved in an extruder or a vessel and transferred using a dosing system to a static mixer, (2) the CO_2_ under elevated pressure is pumped into the same static mixer where both flows are homogenized, and (3) the CO_2_-saturated mixture/solution is depressurized through a nozzle into a spray tower [86,91,92,93]. The nozzle construction enables the formation of fine droplets that become solidified due to the cooling effect of the expanding gas, i.e., polymer particles are precipitated based on the Joule–Thomson effect. High concentrations of sc-CO_2_ in the liquefied mixture/solution lead to a significant reduction of viscosity and interfacial tension.

The RESS process is based on a rapid depressurization of a solution containing sc-CO_2_ and polymer through a specially constructed nozzle into a vessel that maintains lower pressure (usually atmospheric), which leads to a decreased solubility of a polymer, supersaturation, and its precipitation [94,95,96,97]. To improve the solubility of PLA within sc-CO_2_, co-solvents might be necessary. Spherical particles are created if the *M*_w_ of PLA is low enough [98,99].

The SAA process under a reduced pressure can be used to produce microparticles with a defined spherical morphology and controlled particle size [100,101]. SAA implies the formation of a solution (organic solvent and polymer) and its contact for an adequate soaking time with sc-CO_2_ in a saturator to form an expanded liquid, which is characterized by reduced viscosity and surface tension [100]. The formed ternary solution is atomized through a nozzle to produce micro or sub-micro droplets, which upon drying can produce particles of a polymer [87,100]. The atomization is particularly efficient since CO_2_ is released from the internal of the droplets and enhances their fragmentation (formation of secondary droplets). Particles are formed on the basis of a “one droplet one particle” mechanism [102,103]. The difference in particle sizes can depend on the different strength of the cohesive forces operating on the primary droplets (the surface tension and the viscosity). As a rule-of-thumb, the larger the quantity of CO_2_ dissolved in the expanded liquid solution, the higher the reduction of the cohesive forces with the enhancement of the secondary atomization, which is leading to the production of particles with smaller diameters [100,102,103].

Examples of the utilization of CO_2_ as an anti-solvent are processes such as SAS and SEDS. They depend on the diffusivity of sc-CO_2_ into a solution of PLA and an organic solvent. In the SAS process, the organic solution with polymer is sprayed into sc-CO_2_ to cause rapid contact between the two phases [104]. Sc-CO_2_ acts as a solvent for the organic solvent used and as an antisolvent for PLA, which precipitates. The organic solvent is then eliminated from the system along with CO_2_. The choice of the organic solvent for the SAS process is crucial [86], as it may have good miscibility with sc-CO_2_ and PLA at the selected process conditions. PLA micron and submicron particles generated by using SAS are mainly solvent-free. For the SEDS process, the solution containing PLA and sc-CO_2_ is co-introduced into the precipitation chamber through a specially designed coaxial nozzle where sc-CO_2_ acts as both an anti-solvent and “spray enhancer” by mechanical effect [104,105]. The high-velocity turbulent sc-CO_2_ is in favor of the mix and mass transfer, both generating higher super-saturation and prompter precipitation, which results in smaller and finely dispersed particles generated by this method [88]. SEDS can be also run using ultrasound for an additionally enhanced mass transfer (SEDS-EM). It can be applied to generate extremely small droplets, yielding PLA nanoparticles of a uniform shape [106].

As previously described in Section 2.1, the SEE process exploits the solubility of organic solvents in sc-CO_2_, whereby the diffusion and solubility depend on several parameters, including temperature and pressure. By using double emulsions, PLA is dissolved in an organic solvent. While sc-CO_2_ is extracting the solvent, PLA particles containing the suspension are recovered. The final particle size depends on the emulsion droplet size. The SEE process can be run batch-wise or continuously (SEE-C) [42,107,108]. SEE-C allows the improved control of particle size distribution by the reduction of the emulsion processing times and prevention of any droplet/particle aggregation [108].

Table 5 shows a summary of PLA particles prepared via the d-CO_2_ or sc-CO_2_ reported during the last decade. Generally, processes that employ d-CO_2_ for particle generation require less energy and high-pressure equipment of a lower price. However, processes with sc-CO_2_ enable the production of particles with improved properties. It was reported that an increase in process temperature above *T*_c_ for CO_2_ decreases particle agglomeration and increases particles’ uniformity in shape and size [106]. In addition, a pressure increase from below to above *P*_c_ increases the CO_2_ density and reduces the interfacial tension between the solvent and the antisolvent resulting in the reduction of the solution droplet size and consequently the reduction of particles’ size [106]. Besides particle preparation, there are also several reports on modifications to reduce particle size, prevent particle agglomeration, reduce operating pressure and temperature, and improve particle morphology [109,110].

### 4.2. Application of PLA Particles

PLA is approved by the food and drug association agency (FDA) and is one of the most established polymers for application in biomedical and pharmaceutical fields [110], including particle systems. The drugs can be encapsulated in PLA particles or can be attached to the matrix of particles themselves. PLA micro- and nanoparticles have been proposed for improving the bioavailability of poorly water-soluble drugs and as drug carriers for the development of controlled-release devices [115,116,117,118,119]. For instance, it was reported that PLA particles with the anticancer drug 5-fluorouracil and sized from 0.6 to 1.2 μm, obtained by the SAS process, enabled a prolonged and sustained drug release [115]. Besides oral drug delivery, PLA particles can be also developed for subcutaneous and pulmonary delivery [110]. Recently, vaccine vehicles based on PLA nanoparticles as well as the blank PLA microspheres as dermatological fillers for correcting facial wrinkles have received considerable attention [120].

Moreover, porous structures are very accommodative to cell cultures, because the 3D structure closely resembles in vivo conditions. In addition, microspheres of less than 300 μm in diameter undergo a homogeneous degradation with the rate of degradation of the core being equivalent to that at the surface [121]. Thus, PLA microspheres have proven to be useful in facilitating rapid bone growth [122]. In addition, it was reported that fibrous PLA particles obtained using the SAS process are biomaterials suitable for tissue engineering [21,123].

## 5. PLA Impregnation with Bioactive Components Using Sc-CO_2_

**Figure 5:**
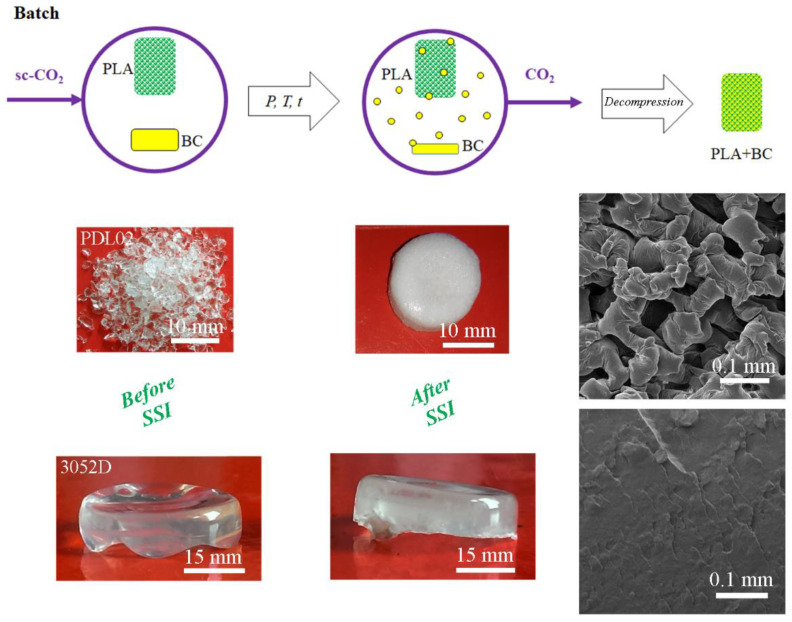
Schematic presentation of the batch SSI process and images of PLA samples before/after SSI with thymol.

**Figure 6:**
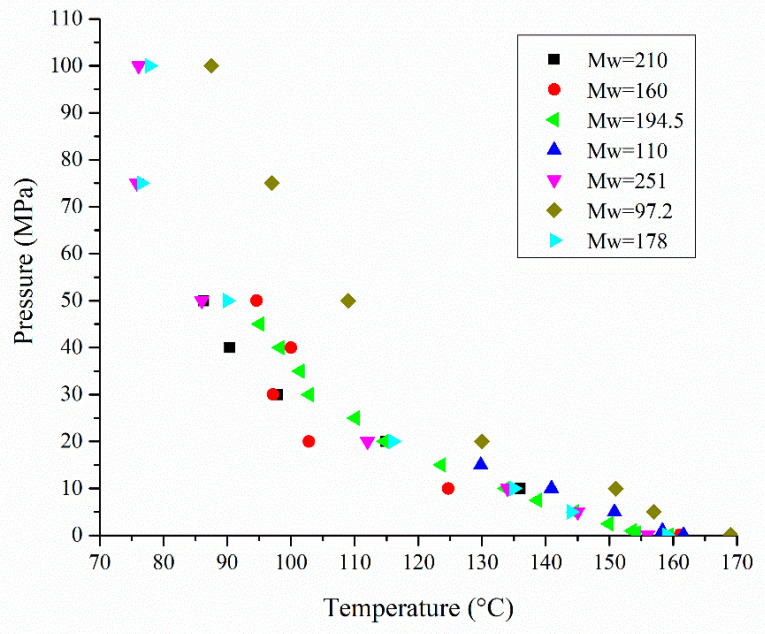
PLA melting point depression in the presence of CO_2_ under elevated pressure and temperature (■—[15]; ●—[15]; ◄—[70]; 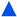
—[138]; ▼—[137]; ♦—[137]; ►—[137]).

The impregnation process under an elevated pressure implies the exposure of polymeric material to a supercritical solution, i.e., sc-CO_2_ and a certain substance, usually a single bioactive component (BC). This process requires good solubility of the substance in question in sc-CO_2_, which facilitates its mass transport within the polymeric matrix [18]. If the solubility of a BC in sc-CO_2_ is low for the PLA impregnation process, it could be improved by the addition of a polar co-solvent such as ethanol [124,125]. After contact between a polymeric material and a BC for a certain soaking time, sc-CO_2_ can be completely removed from the obtained material by a pressure decrease, while BC will remain within the polymer matrix due to precipitation or chemical bonding [86,126]. The supercritical solvent impregnation process (SSI) has significant advantages compared to conventional impregnation processes associated with sc-CO_2_ properties. It allows the impregnation of polymeric materials with a variety of BCs including hydrophobic molecules such as essential oils [127]. This technique can improve polymer impregnation due to the low viscosity and good transport properties of sc-CO_2_ as well as the high solvent power shown to organic molecules at moderate temperatures [4,126]. For instance, Trindade Coutinho et al. [128] reported that the SSI process of 3 h enabled 2.6 times higher drug loading into PLA film compared with the conventional soaking method that lasted 10 days. An additional advantage of SSI is that the BCs’ load and distribution through a polymer matrix can be easily tuned by changing the process conditions [15,25,129]. When sc-CO_2_ leads to PLA plasticization and swelling, enabling the mobility of macromolecular chains and increased space, it favors the sorption of BCs [14,18,25]. In addition, the SSI technique allows work at relatively mild conditions in an oxygen-free environment that is often desirable for sensitive BCs [127,129]. BCs can be loaded into various polymeric forms such as porous and non-porous films, monoliths, scaffolds, aerogels, particles, or fibers using sc-CO_2_. It was reported that the process of PLA impregnation with BCs can be performed using sc-CO_2_ in a batch or a semi-continuous regime [14]. In the batch process, PLA and BC are placed in the same vessel with or without the provided mixing or circulation of the supercritical fluid solution (sc-CO_2_+BC) (Figure 5). The semi-continuous impregnation involves the subsequent introduction of fresh CO_2_ into the vessel, dissolution of additional amount of BC in sc-CO_2_, and flow of the supercritical solution through PLA (Figure 6).

### 5.1. Methods for PLA Impregnation Using Sc-CO_2_

The overview of the SSI process performed on PLA materials in various forms is shown in Table 6. Reports from the available literature indicate that the SSI process with sc-CO_2_ allows the homogenous distribution of BC through the PLA matrix [130,131]. This is primarily attributed to the interaction of BC and PLA through the hydrogen bonding between the carbonyl groups of polyester and the hydroxyl groups of BC [14,132].

Besides the good solubility of BC in sc-CO_2_, one of the main criteria for achieving high loadings is the good affinity between PLA and BC, as well as the selection of proper processing conditions [133]. The amount of incorporated BC into PLA can be controlled by variations in pressure and temperature. Usually, an increase in pressure increases BC loading, while an increase in temperature decreases BC loading [75,134]. However, it was also reported that BC loading into PLA can be increased both with pressure and temperature [20,125,129,135,136]. Considering that the pressure and temperature determine CO_2_ density and its solvation power, this result can be obtained when the crossover pressure for BC dissolution in sc-CO_2_ is reached/overcome [136]. It was reported that BC loading can be predicted mainly through the study of the phase behavior between BCs and sc-CO_2_ [126]. An increase in BC loading with both the pressure and temperature can be also the result of changes in the PLA morphology during the SSI process. Namely, the CO_2_ sorption and PLA swelling increase as the process temperature increases [136], as it gets closer to the in situ *T*_m_ of PLA [20,75,129,133]. In addition, it was also reported that an increase in pressure induces a depression of *T*_g_ and *T*_m_, confirming that d-CO_2_ and sc-CO_2_ have a plasticizing effect on PLA [15]. As illustrated in Figure 6, the *T*_m_ of several types of PLA continually decreases when the pressure increases [15,137,138]. A greater variation in *T*_m_ depression for PLA with different *M*_w_ is observed at higher CO_2_ pressures.

Usually, an increase in soaking time increases the BC loading into the PLA matrix [15,75,125,139]. Kuska et al. [15] reported an increase in thymol loading into PLA beads from 11.0 to 19.8% at 20 MPa and 120 °C by prolongation of the process from 2 to 24 h. This BC acted as a molecular lubricant, which resulted in the increased free volume of the PLA matrix and consequently in the higher sorption of a supercritical solution. The prolonged exposure of PLA to sc-CO_2_ with thymol led to a 40–90% greater swelling extent in comparison to the first 2 h. A similar result was reported for the SSI of olive leaves extract into PLA filaments, where the prolongation in soaking time from 0.5 to 1 h increased both the swelling and loading [125]. However, an increase in the soaking time can also lead to a decrease in the loading of BC during the SSI process. Namely, it was reported that a longer processing time (15 h) led to a significant decrease in the thymol loading into PLA films due to the increased crystallinity of the polyester matrix saturated with sc-CO_2_ over time [14]. The lowering of the amorphous phase and free volume of the polymer matrix consequently resulted in the lower sorption of sc-CO_2_ and thymol in the polymer [14].

The decompression rate is an additional parameter that significantly affects the loading of BCs into the PLA matrix. Mainly, a decrease in the decompression rate increases the BC loading. For instance, an increase in ketoprofen loading into PLLA sutures from 11.8 to 19.8% was reported for a decrease in the decompression rate from 360 to 0.06 MPa/min for SSI performed at 30 MPa and 80 °C during 3 h [20]. Moreover, thymol loading into the PLA film increased from 14.7 to 20.4% for a decrease in the decompression rate from 10.0 to 0.1 MPa/min for SSI performed at 12 MPa and 40 °C during 3 h [4]. Namely, higher depressurization rates involve lower diffusion coefficient values, which were explained by a slight modification of the arrangement of the polymeric chains due to the rapid expansion at the last step of the impregnation processing [4]. On the other hand, Villegas et al. [127] reported that a variation in the decompression rate from 0.1 to 10 MPa/min at a pressure of 9 or 12 MPa was not significantly relevant for the amount of cinnamaldehyde incorporated into the PLA films, while it strongly influenced the film mechanical properties. The lowest decompression rate leads to an increase in the elongation at break and a decrease in the tensile modulus and strength of PLA films [127].

**Table 6:** Literature reports on PLA impregnation using sc-CO_2_ (2012–2022).

PLA	PLA Form	Bioactive Component	*P* (MPa)	*T* (°C)	*t* (h)	Dec. (MPa/min)	Material Properties	Material Application	Lit
2003D	Film	Thymol	9, 12	40	3	0.1, 1, 10	*L* = 13–20%; *σ* = 13–21 MPa *T*_m_ = 130–141 °C; *χ* = 1.2–3.3%	Active food packaging	[4]
PLA	Filament	Ketoprofen	10–40	35–75	2	Fast	*L* = 0.1–9.0% *Sw* = 4–30%	Biomedical applications	[135,136]
2003D	Beads	Thymol	20–30	100–120	2, 24	180	*L* = 4.7–19.8%; *Sw* = 11–47% *ρ* ~470 kg/m^3^; *ε*~61%	Bioactive materials	[15]
PLLA	Suture fibers	Ketoprofen, aspirin	10–35	40–140	3	0.06, 6, 360	*L* = 0.5–32%; *T*_m_ = 155–170 °C	Bioactive sutures	[20]
PLA	Films	Multiflora extract	15, 25	45, 65	2, 8	0.5	*L* = 6.7–23.8%; *T*_m_ = 167.8 °C; IC_50_ = 19.9 μg/mL	Active food packaging	[126]
2003D 3052D	Beads	Thyme extract	30	110	2–7	180	*L* = 0.2–0.7%; *ε* = 64–76% *ρ* = 299.4–433.2 kg/m^3^	Bioactive materials	[15]
PLLA	Fibers	Ketoprofen	30, 35	80, 90	3	0.06, fast	*L* = 12–33%; *DR* = 3 days–3 months	Bioactive sutures	[129]
PDL02	Flakes	Thymol	10, 15	35–50	2–24	30	*L* = 2.3–18.8%; *T*_g_ = 32.4 °C; *D*_aver_ ~51.4 μm; *DR* = 1 month	Biomedical devices	[75]
PLA	Filaments	Mango extract	10.1, 25.3, 40.5	35–55	1–3	3	*L* = 1–9%; *OI* = 21–90%; IC_50_ = 8.24 μg/mL; *ADC* = 4.3–25.0%	Bioactive materials	[134]
PLLA	Films	Aspirin, carvone, ketoprofen	9, 30	60, 80	3	Fast	*L* = 1.4–26.5%; *DR* = 20 days–2 months	Drug delivery	[128,133]
PLA	Films	R-carvone	8–17.8	40, 60	2	0.6, 6.0	*L* = 6.7–29.8%; *T*_g_ = 40.4 °C; *T*_m_ = 133.0 °C; *χ* = 25.7%	Active food packaging	[139]
3052D	Disc, film	Thymol	10	40	2–24	n.i.a.	*L* = 6–30%; *Sw* = 0.1–7.5%	Active food packaging	[140]
2003D	Film	Cinnamaldehyde	9, 12	40	3	0.1, 1, 10	*L* = 8–13%; *χ* = 0.6–3.1%; *σ* = 14.3–38.0 MPa	Active food packaging	[127]
PLA	Filaments discs	Mango leaf extract	10, 40	35, 55	2	4, 10	n.i.a	Endoprostheses	[141]

*P*—pressure; *T*—temperature; *t*—time; *L*—bioactive component loading; *Sw*—swelling extent; *D*_aver_—average pore diameter; *ε*—porosity; *σ*—tensile strength; *T*_m_—melting temperature; *χ*—crystallinity; *ρ*—foam density; *ε*− porosity; IC_50_—concentration to scavenge 50% of DPPH radicals; *DR*− drug release; *OI*—oxidation inhibition; *ADC*—antidenaturant activity; *D*—diameter; n.i.a.—no information available.

Based on the literature reports, it can be concluded that the SSI process is complex and that it is affected by numerous parameters. Namely, the loading of BCs into PLA using sc-CO_2_ is controlled by: (1) properties on neat PLA and BC, (2) the potential swelling and plasticizing effect of sc-CO_2_, (3) the initial ratio of PLA/BC, sc-CO_2_/BC, and sc-CO_2_/PLA/BC, (4) the solubility of BC in sc-CO_2_, (5) the affinity of BC towards PLA, (6) the interaction of PLA with BC, (7) the process pressure, (8) the temperature, (9) the contact time, (10) the decompression rate, and (11) the addition of a co-solvent.

### 5.2. Application of PLA Material Impregnated with Bioactive Substances

PLA impregnated using sc-CO_2_ has been actively investigated for the development of active food packaging. These active materials are the driving force for innovation, as PLA is minimally processed in an environmentally friendly manner, and it can extend food shelf life, enhance food safety and/or sensory properties, and contribute to the preservation of food quality [58,126,127]. In addition, these materials can be recycled or they can degrade in the environment after disposal [139]. Villegas et al. [127] demonstrated that PLA films impregnated with thymol and cinnamaldehyde are active against *Escherichia coli* and *Staphylococcus aureus* and could be potentially used for food packaging.

PLA materials impregnated with BCs by sc-CO_2_ can be used for the preparation of drug delivery systems [66,133]. For instance, PLA was impregnated with aspirin used as an anti-inflammatory drug [128], triflusal used in the prevention of cardiovascular events [66], and paclitaxel used in cancer therapy [124]. In addition, sc-CO_2_ has been widely used for the preparation of bioactive medical devices such as intraocular lenses, sutures, and scaffolds for tissue engineering. PLA-BC sutures can be used in surgery for wound closing or for a prosthesis to reduce pain, inflammation, and infection at the surgery site, as well as for oral drug administration [20,130]. This local delivery of drugs can limit drug side effects, provide bioactivity at site-specific places, and increase patient adherence [20]. Three-dimensional-printed filaments of PLA were impregnated with ethanolic olive leaves extracted for biomedical application [125]. These filaments demonstrated a 2,2′-diphenyl-1-picrylhydrazyl (DPPH) radical inhibition of 6–15% for the antioxidant loading of 0.2–0.5 mg/g, which can be used for a decrease in free radicals that induce oxidative damage in biomolecules (e.g., lipids and proteins).

## 6. Emerging Sc-CO_2_ Techniques for PLA Processing

### 6.1. Integrated Processes of Supercritical Extraction of Bioactive Compounds and PLA Impregnation

**Figure 7:**
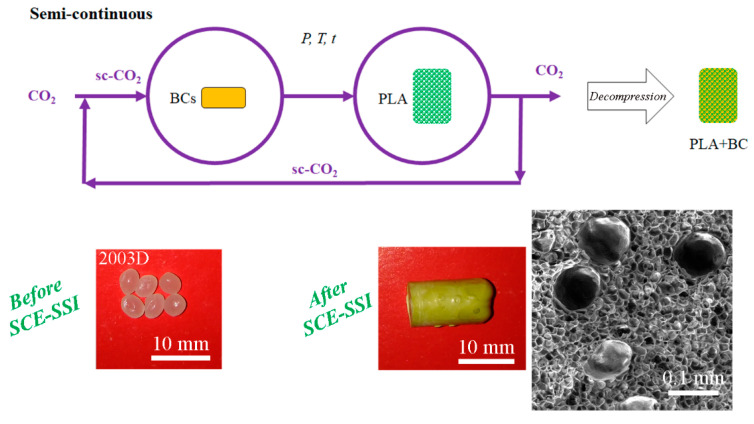
Schematic presentation of the semi-continuous SSI process and images of PLA samples before/after SCE-SSI of thyme extract.

Integrated processes of supercritical extraction and impregnation (SCE-SSI) imply the coupling of extraction from the plant material and the impregnation of polymeric material with plant extract using sc-CO_2_ without the intermediate step of decompression. The main challenge in harmonizing the SCE-SSI process is the synchronization of the extraction and impregnation rates by choosing the adequate processing mode regarding the circulation of CO_2_ and processing time to achieve a maximum extraction and impregnation yield, preventing the desorption of already impregnated extract from the carrier [142]. This integrated process minimizes the extract loss in the tubes and vessels of the high-pressure equipment, as well as the processing time and energy consumption [14,15,142,143]. Additionally, the exposure of a valuable bioactive extract to the air and light is avoided [143]. It can be performed in a semi-continuous regime (Figure 7). To the best of our knowledge, there are only two reports in the available literature on the application of the SCE-SSI process for the functionalization of PLA materials [14,15]. Kuska et al. [15] applied the integrated SCE-SSI for the separation of extract thyme and its impregnation into PLA at 30 MPa and 110 °C. It was reported that thyme extract does not interact chemically with the PLA matrix and that an operating time longer than 2 h had a negative effect on the thyme extract loading due to the desorption of the already impregnated extract from the PLA matrix. To increase the loading of thyme extract, the introduction of fresh CO_2_ into the system was proposed (after 2 h of the supercritical solution circulation, the circulation was stopped, and only the adsorber was depressurized and then refilled and re-pressurized with fresh CO_2_). The refill of fresh CO_2_ led to a three-fold higher loading after an additional 2 h of impregnation. Therefore, it can be hypothesized that, besides the previously mentioned parameters that affect the batch SSI process of PLA, the number of cycles for the introduction of fresh CO_2_ into the system also have an impact on BC loading.

### 6.2. Deposition of Metal Oxides onto PLA

**Figure 8:**
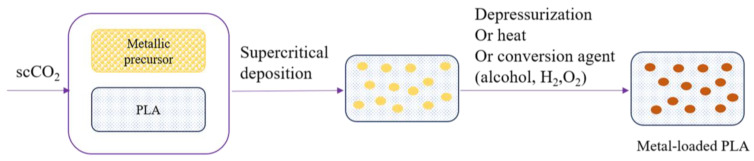
Schematic presentation of supercritical fluid deposition.

Supercritical fluid deposition (SCFD) using sc-CO_2_ is an attractive alternative process for the controlled dispersion of metallic species onto or within the surface of porous solid polymers [144]. This technique consists of three steps (Figure 8): (1) the dissolution of a metal complex (precursor) in a supercritical fluid, (2) its adsorption onto a polymer matrix, and (3) the conversion of the adsorbed complex to the target metal species [144,145,146]. Precursors usually used in SCFD are metal chelates, fluorinated metal chelates, and organometallic complexes soluble in sc-CO_2_ [145]. Their solubility and phase behavior in sc-CO_2_ are important parameters, since the following adsorption process depends on their concentration in the fluid phase. The sc-CO_2_ solvent power and sorption of the metal complex can be adjusted by pressure and temperature change as well as the contact time of sc-CO_2_ with a metal complex solution and a polymer [144]. Once a polymer–metal precursor composite is prepared, the metal precursor can be converted to its metal form using different methods: chemical conversion at low pressure after the depressurization process, chemical reduction using reducing agents such as alcohol, H_2_, O_2_, or by a thermal reduction in sc-CO_2_, or thermal decomposition at a low pressure in an inert atmosphere [144,145,147]. Porous PLA-based composite materials were usually synthesized using common methods such as solvent casting, salt leaching, injection molding, 3D printing, phase separation, electrospinning, and freeze-drying. However, the only study to date on SCFD onto PLA was reported by Ivanovic et al. [144]. The authors reported that the sc-CO_2_-assisted deposition combined with the PLA foaming process, followed by the in situ synthesis of ZnO, was carried out for the preparation of composite scaffolds. They used sc-CO_2_ both as a physical blowing agent and medium for the deposition of organometallic precursors in a single step-process performed at a pressure of 30 MPa and temperature of 110 °C. Zinc bis(2-thenoyltrifluoroacetonate) was used as a ZnO precursor, and scaffolds impregnated with the precursor were treated with hydrazine solution in absolute ethanol for the chemical conversion. The applied procedure provided a good interaction of ZnO with the polymer substrate, a positive effect on the rearrangements of the crystalline structure, and improved compressive strength.

Although SCFD was mainly used for the production of nanostructured materials for catalysis, electronics, and optics, its great potential for the fabrication of medical devices has also been recognized as the possibility of the sc-CO_2_-aided deposition of metal species with antibacterial activity onto different carriers [144]. The development of composites is one of the major methods to address some of the problems associated with PLA use in biomedicine, such as low cell adhesion, biological inertness, low degradation rate, and acid degradation by-products [148]. The incorporation of nano/micro inorganic fillers (e.g., clays, talc, apatite, and carbon) can improve the mechanical and bioactive properties of polymeric scaffolds, while the incorporation of metals and their oxides (e.g., Zn, Ag, Ca, Mg, and Ti) can provide antimicrobial activity [144]. Moreover, the presence of metallic nanofillers in/on PLA can provide a high thermal conductivity, increase degradation, improve surface roughness, and enhance cell adhesion [148]. There are numerous advantages in the preparation of PLA/metal nanocomposites using this supercritical approach. Specifically, the high sc-CO_2_ diffusivity and the ability to swell the polymeric substrates, enabling the high penetration of organometallic reagents, as well as the outstanding control of the composites’ morphology, coupled with a good solubility of a wide range of possible precursors in sc-CO_2_, promote the SCFD as a highly promising technique for the design of novel systems with tunable properties for tissue engineering applications.

## 7. Future Perspectives

PLA materials are already the number one choice for producers that are working towards sustainable production and for consumers that demand biocompatible and/or biodegradable products. In addition, the future of the commercialization of high-pressure CO_2_ technologies for PLA processing looks bright, as it allows for the preparation of small and large-scale materials with precise and superior properties. However, the diverse composition and processing behavior of PLA, as well as various processing techniques combined with numerous process parameters and a lack of systematic characterization data, are accountable for the slow progress in technology and product development.

The investigation of high-pressure CO_2_ technologies for the processing of PLA materials and especially the production of micro- and nanoparticles for biomedical applications is expected to expand. The pursuit of nanoscale porous PLA materials characterized by a 3D structure represents a new realm of research for functional materials design and fabrication. Using the 3D printing process for the production of new forms of biocompatible polymers can provide personalized devices that are efficient, safe, and precisely tailored to the patient’s compliance. Considering that high temperatures are employed for the 3D printing process, making it inapplicable for thermolabile bioactive compounds, the SSI process can emerge as a contributing process for enduing printed devices with bioactivity. There are few recent publications describing the 3D printing of PLA filaments and their impregnation with drugs and plant extracts. However, a combination of the mentioned processes can still be considered emerging. The technological combination of 3D printing technology and supercritical drying to produce 3D-printed aerogels could help solve the lack of the as-printed material nanostructuration, which is one of the current 3D printing limitations [149,150]. This strategy should enable obtaining biomaterials with improved performance and architecture or enhanced properties regarding their degradation behavior in biological fluids, mechanical integrity, and sterility to be compliant with regulations for implantable medical devices [149,150].

One of the technologies that could be combined with high-pressure CO_2_ technologies is the laser-based additive manufacturing of polymer components, which is an established process in the automotive industry, among others. Unfortunately, most applications are limited to polyamides, as other polymers are hardly offered as suitable particle systems [151]. Sc-CO_2_ can be used to produce spherical particles from other polymers in the size range required for additive manufacturing. PLA can also be converted, for example using the PGSS process, into a flowable powder that can be used for powder bed processes or for laser deposition processes to produce 3D components. Together with other powdered materials, additive manufacturing can also be used to produce hybrid components that cannot be created using classic manufacturing processes.

Blending PLA with other biopolymers or plant-based materials is an area of active research for improving the properties of PLA. Blending PLA with other biopolymers, such as starch, cellulose, or chitin, can improve the material’s mechanical properties, moisture barrier, and biodegradability. Bends can also reduce the brittle nature of pure PLA, which can improve its impact resistance. Another area of research is the use of plant-based materials, such as fibers or particles, as fillers in PLA composites. This can lead to improved strength and stiffness, as well as a reduced weight and cost. Using plant-based materials in PLA composites also can make the materials more sustainable and environmentally friendly. It is worth mentioning that the compatibility between different polymers, the dispersion, and the interphase adhesion are the main challenges that need to be overcome when working with biopolymer blends. Overall, blending PLA with other biopolymers or plant-based materials is a promising area of research for improving the properties and sustainability of PLA materials. With continuing development in these areas, it is expected that more advanced and functional materials can be produced using PLA and other biopolymers.

The reuse of long-life PLA products is possible; however, PLA is mainly used for the production of short-life products. To make PLA into a low-carbon material, the development of recycling facilities is necessary. A life cycle assessment study demonstrated that the recycling of PLA waste supports the reduction of environmental impacts [152]. All investigated recycling alternatives, namely mechanical, solvent-based, and chemical, were proved to be suitable and show clear environmental benefits. Besides the environmental benefits achieved by replacing the virgin PLA with PLA recyclates, it is also economically more favorable, since recyclates are sold for approximately two times a lower price compared to the primary material [152]. During the treatment of PLA waste, the material is exposed to external influences, which may cause physical alterations in the color and molecular masses, or result in the inclusion of impurities; hence, the generated products can have a lower quality than virgin PLA products [152]. The application of high-pressure CO_2_ processing technologies to obtain new PLA products from recycled PLA materials, which will maintain good quality and acceptable properties, may also be a good direction towards the increased PLA sustainability.

Overall, despite a large number of scientific papers, the obvious advantages of sc-CO_2_ for the processing of PLA, and a wide range of potential applications of the obtained materials with improved properties, the research on using the supercritical fluid processing for PLA is limited and has not yet reached a commercial scale. The main challenges for industrial supercritical fluid processing are cost, energy requirements, and scalability. However, the development of new techniques, such as the continuous supercritical fluid processing and the decreasing cost of CO_2_ storage, may help overcome these barriers and make it more viable for commercial production. Therefore, with the future development of newer technologies and cost-effective methodologies, supercritical fluid processing could become a promising technique for the production of advanced PLA materials.

## 8. Conclusions

This review summarized state of the art on the processing of neat PLA using an environmentally friendly medium (i.e., dense and supercritical CO_2_). It was demonstrated that easily tunable properties of CO_2_ under an elevated pressure could be employed for the development of advanced PLA materials that can find a variety of applications from thermal insulation to biomedicine.

Dense and supercritical CO_2_ can be successfully used for PLA material drying, foaming, particle generation, and impregnation processes. The review sums up the complexity of all of these processes by pinpointing the next parameters as crucial: (1) properties on neat PLA (melting temperature, glass transition temperature, crystallinity, etc.), (2) process pressure, (3) process temperature, (4) process operating time, (5) process decompression rate, (6) process regime (static, semi-continual, and dynamic), (7) effect of d-CO_2_/sc-CO_2_ on PLA (potential swelling and plasticizing effect), (8) extrusion parameters (barrel temperature, number of temperature zones, number of screws, design of screws, screw speed, torque, blowing agent inlet, die temperature, and die design), (9) selection of PLA solvent, (11) initial ratio of PLA and solvent, (12) addition of porogen or antisolvent, (13) initial ratio of components in the system (PLA vs. d-CO_2_/sc-CO_2_, PLA vs. bioactive component, d-CO_2_/sc-CO_2_ vs. bioactive component), (14) behavior of ternary system (d-CO_2_/sc-CO_2_+PLA+bioactive component), (15) solubility of bioactive component in d-CO_2_/sc-CO_2_, (16) affinity of bioactive component towards PLA, (17) chemical interaction of PLA with bioactive component, (18) number of cycles for the introduction of fresh CO_2_ into the system, and (19) addition of a co-solvent. The mentioned parameters should be investigated and/or optimized for every individual CO_2_-PLA system and process under an elevated pressure to improve the end product’s performance and develop superior PLA materials with controlled properties for advanced applications.

This review could stimulate the further development of PLA processing using CO_2_-assisted techniques that may open a wide range of opportunities for designing novel, functional materials/products and drive the field forward in the green future.

## Figures and Tables

**Table 1:** Leading PLA producers and processability/application possibilities of their PLA.

Company	Industrial Name	Intended Processing Methods	Applications
NatureWorks (Plymouth, MN, USA)	Ingeo^®^	- Extrusion - Thermoforming - Injection molding - Films and cards - Three-dimensional printing - Fibers and nonwovens - Foaming	- Beauty and household - Building and construction - Cartons and non-food packaging - Electronics and appliances - Food and beverage packaging - Food service ware - Medical and hygiene - Landscape and agriculture
TotalEnergies Corbion (Gorinchem, The Netherlands)	Luminy^®^	- High heat - Demanding applications - General purpose applications - Seal layer	- Rigid food packaging - Flexible packaging - Fresh food packaging - Durable goods - Non-wovens - Food service ware
Evonik Industries (Essen, Germany)	Resomer^®^	- Three-dimensional printing - Fused filament fabrication	- Bioresorbable medical implants - Controlled release devices
ThyssenKrupp (Essen, Germany)	PLA*neo*^®^	- Extrusion - Injection-molding	- Packaging material - Films and plastic bottles - Textile materials such

**Table 2:** Technologies that employ d-CO_2_ and sc-CO_2_ for PLA processing to obtain advanced functional products.

Technique	Product	Products’ Main Properties
Drying	Aerogels Scaffolds	Low density High porosity High specific surface area
Foaming	Foams	Highly porous structure
Particle generation	Microparticles Nanoparticles	Tunable size Controlled particle size distribution
Impregnation	Variety of forms	Material with organic or inorganic compounds Active material (bio or photocatalytic activity)

**Table 5:** Literature reports on PLA particle generation using d-CO_2_ and sc-CO_2_ (2012–2022).

PLA	Producer	Solvent	Method	*P* (MPa)	*T* (°C)	Particle Size	Application	Lit
PLLA, PDLA	Synthetized	DME, chloroform	Static PGSS	10	60–100	*d* = 1.1–11.6 µm	Filler in composites	[111]
PLA	Sigma-Aldrich	Ethanol, acetone, DCM	RESS	27.5	35	*d* = 2–3 µm	Drug delivery	[99]
PLLA	Sigma-Aldrich	DCM, TCM, methanol, acetone	RESS, SAS	15–30 10–25	45–100 35	*d*_RESS_ = n.i.a *d*_SAS_ = 1–104 µm	Drug delivery	[112]
PLLA	Sigma-Aldrich	DCM	SAS	n.i.a.	35–50	*d* = 0.6–47.3 µm	Biomedical applications	[113]
PLLA	Purac	DCM	SAS	8–16	40	*d* = 5–25 µm	Veterinary applications	[101]
PLLA	Sigma-Aldrich	DCM	SAS	10–20	40	*d* = 0.08–1.43 µm	Drug delivery	[114]
PLA	Synthetized	DCM	SEDS-EM	7.9–9.8	24–38	*d* < 400–1000 nm	Controlled release	[106]
PLA	Evonik	Ethylacetate	SEE-C	8	38	*d* = 203 nm–0.9 µm	Bactericidal nanocomposites	[42]
PLA	Boehringer	Acetone	SEE-C	8	38	*d* = 212–233 nm	Controlled release	[108]
PLLA	Sigma-Aldrich	DMSO+DCM	SAS	18	50	*d* < 1 µm	Drug delivery	[115]

## Data Availability

Not applicable.
